# Sensitivity to Aortic Rupture in Hereditary Aortic Diseases

**DOI:** 10.1055/a-2378-9201

**Published:** 2024-08-31

**Authors:** Vivian de Waard

**Affiliations:** 1 Department of Medical Biochemistry, Amsterdam UMC Locatie AMC, Amsterdam, Noord-Holland, The Netherlands

Novel Insights into the Aortic Mechanical Properties of Mice Modeling Hereditary Aortic Diseases

In different hereditary aortic diseases (hADs), aortic dissection/rupture is the main event to focus on to improve morbidity and mortality. Sometimes this is preceded by aortic aneurysm formation, but often this is not the case. It demonstrates that aneurysm formation and aortic rupture are not necessarily the same process. In this issue of *Thrombosis and Haemostasis* , the manuscript from Dubacher et al showed that these processes are separated, 1 shedding new light on what is essential for aortic rupture.

The authors mounted aortic rings derived from different locations within the thoracic aorta, and taken from six murine models with genetic variants related to hAD, on a tissue puller and uniaxially stretched the aorta until rupture, measuring aortic diameters and tensile force. A reduced aortic rupture force signifies compromised aortic extracellular matrix (ECM) integrity. Thus, as expected, the aortic rupture force of mice with a collagen-3 defect ( *Col3a1* ^m1Lsmi^ ), representing vascular Ehlers–Danlos syndrome (vEDS), was low when compared with wild-type mice. Also, both *Fbn1* ^C1041G/+^ and *Fbn1* ^mgR/mgR^ models of Marfan syndrome (MFS) showed reduced rupture force, but not as much as the vEDS aortas. These hAD mice showed signs of impaired aortic integrity at the age of euthanasia. In vEDS patients, arterial rupture often occurs without aneurysm formation, which is similar in this model, since no aortic diameter differences were observed upon stretch in vEDS aortas versus wild type. In MFS patients, aortic dissections/rupture is mostly preceded by aortic aneurysm formation in the ascending aorta, making it easier to determine timing for vascular surgery in MFS. Interestingly, the *Fbn1* ^C1041G/+^ mice did not have enhanced aortic stretch yet, while the *Fbn1* ^mgR/mgR^ mice did. Despite this difference in aortic stretch, both MFS lines were prone to aortic rupture.

Of these MFS models, the *Fbn1* ^C1041G/+^ mice normally develop aneurysms, but due to their fibrotic medial thickening phenotype, these aneurysms do not rupture. However, the more severe *Fbn1* ^mgR/mgR^ MFS model shows aortic thinning, aneurysm development, and is prone to spontaneous rupture. Thus, the *Fbn1* ^mgR/mgR^ MFS model was used for an intervention study to assess if the angiotensin-II receptor type 1 blocker (ARB) losartan could improve the aortic rupture force. The 4-week losartan treatment did reduce aortic stretch in the ascending aorta, but surprisingly did not impact the aortic rupture force. This suggests that the inherent ECM defect responsible for enhanced rupture risk is not repaired by (short-term) ARB treatment, while aneurysm formation is.

**Fig. 1:**
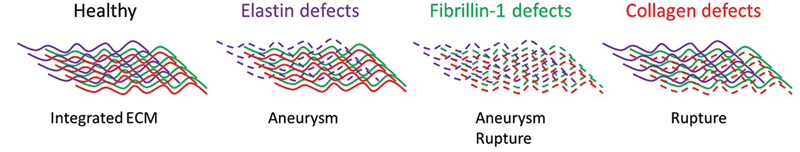
The ECM in the aorta consists for the largest part of three types of fibers, namely elastic (also containing fibrillin-1), fibrillin-1, and collagen fibers, which are all connected to function as one entity. When different components of this network are defective, it impacts the aortic biomechanics. It seems that aneurysm development and rupture risk are dependent on different ECM components. The stretch and rupture force experiments point at elastin defects causing aortic stretch (aneurysm) and collagen defects causing aortic rupture, while fibrillin-1 defects can cause both, thus being important for the integrity and/or connectivity of the entire ECM network. ECM, extracellular matrix.

Fibrillin-1 encoded by FBN1 is an ECM protein forming large fibers. It can form an independent network integrated in the ECM or be used as a template for elastin sheets/fibers in elastic tissues. It also sequesters growth factors such as the different transforming growth factor β (TGFβ) family members, necessary for growth and wound healing. So fibrillin-1 can serve mechanosensing and signaling roles within one tissue. The MFS data here reveal that while elastin integrity is preserved with reduced aortic stretch upon ARB treatment, the aorta is still at risk for rupture. Since the mild MFS model actually showed a similar profile as the vEDS aortas, with no stretch but reduced rupture force, it thus points at a role for fibrillin-1 integration with the collagen network ( Fig. 1 ). Along those lines, it was already demonstrated that the collagen network in the MFS patient aorta is less integrated. With atomic force microscopy, using different size probes for indentation, it showed that the resistance was similar in control tissue independent of probe size, while this separated in MFS tissue, revealing loss of ECM crosslinking. 2 Moreover, in MFS patients the type A dissections in the ascending aorta mostly coincide with aneurysm formation; however, type B dissections that occur in the thoracic descending aorta do not. 3 Also, FBN1 variants are found that just cause aortic/arterial dissection without classification for MFS. 4 5 6 Clearly, aneurysm formation is not a requirement for dissection or rupture.

Furthermore, regional aortic differences were observed between the aortas of MFS mice and smooth muscle cell (SMC)-specific Efemp2 (fibulin-4)-deficient mice, resembling cutis laxa. Fibulin-4 is involved in elastin and collagen fiber assembly and crosslinking in the ECM. 7 In the MFS mice, the aortic rupture force was reduced throughout the thoracic aorta, while in the SMC-Efemp2-deficient mice, it was only reduced in the ascending aorta, localizing rupture-prone sites.

This system is also very informative for analysis of novel variants of unknown significance. Here, three potentially interesting hAD variants in Ltbp1 (TGFβ transport to and sequestering in the ECM and fiber assembly), Mfap4 (fibrillin/elastin fiber assembly), and Timp1 (inhibitor of matrix metalloproteinases to protect ECM from degradation) were mimicked in mice and tested. For MFAP4, it has been shown that high plasma MFAP4 in MFS patients is associated with type B aortic dissection. 8 These genes are currently not known as hAD genes, however related genes are, such as LTBP3 and MFAP5 variants. None of the mice with either heterozygous or homozygous mutations showed susceptibility to aortic stretch or rupture, even at an old age. This suggests that these tested variants are benign.

Often there are sex differences observed in disease severity in hAD, which was most prominent here in the MFS *Fbn1* ^mgR/mgR^ model, where the females showed slightly less aortopathy. This is also known for MFS patients when studying large cohorts. 9 In the females, losartan could rescue aortic stretch better than in males.

In conclusion, the technique applied here allows distinguishing between aneurysm risk and rupture risk, and will answer important questions such as regional aortic sensitivity to dilation or rupture, likelihood of a variant of unknown significance to have a significant impact, sex differences or drug efficacy in improving either aortic dilation, rupture risk or both. Awareness of potential different processes responsible for aneurysm formation or dissection/rupture may shift the research focus to other types of biomarkers, imaging tools, and therapeutics, 10 and will broaden our horizon of hAD diagnosis and management.
